# Antibody Recognition of Shiga Toxins (Stxs): Computational Identification of the Epitopes of Stx2 Subunit A to the Antibodies 11E10 and S2C4

**DOI:** 10.1371/journal.pone.0088191

**Published:** 2014-02-05

**Authors:** Yongjun Jiao, Fiona S. Legge, Xiaoyan Zeng, Herbert R. Treutlein, Jun Zeng

**Affiliations:** 1 Institute of Pathogenic Microbiology, Jiangsu Provincial Center for Disease Prevention and Control, Key Laboratory of Enteric Pathogenic Microbiology, Ministry Health, Nanjing, China; 2 Monash Institute of Pharmaceutical Sciences, Monash University, Parkville, Victoria, Australia; 3 Computist Bio-Nanotech, Small Technology Clusters, Scoresby, Victoria, Australia; National Cancer Institute, NIH, United States of America

## Abstract

We have recently developed a new method to predict the epitopes of the antigens that are recognized by a specific antibody. In this work, we applied the method to identify the epitopes of the Shiga toxin (Stx2 subunit A) that were bound by two specific antibodies 11E10 and S2C4. The predicted epitopes of Stx2 binding to the antibody 11E10 resembles the recognition surface constructed by the regions of Stx2 identified experimentally. For the S2C4, our results indicate that the antibody recognizes the Stx2 at two different regions on the protein surface. The first region (residues 246-254: ARSVRAVNE) is similar to the recognition region of the 11E10, while the second region is formed by two epitopes. The second region is particularly significant because it includes the amino acid sequence region that is diverse between Stx2 and other Stx (residues 176-188: QREFRQALSETAPV). This new recognition region is believed to play an important role in the experimentally observed selectivity of S2C4 to the Stx2.

## Introduction

*Escherichia coli* O157:H7 and other Shiga toxin (Stx)-producing *E. coli* (STEC) strains cause over 10000 infections and over 90 deaths each year in the United States [1]. In China, it was responsible for two large disease outbreaks in three neighboring Provinces (Jiangsu, Anhui and Henan) between 1999–2000. The infection with STEC causes diarrhea and hemorrhagic colitis, and potential development of hemolytic-uremic syndrome (HUS) characterized by acute renal failure, thrombocytopenia, microangiopathic hemolytic anemia, and death [2].

The Shiga toxins consist of a single domain A-subunit and a pentamer B-subunit. The 32 kDa A-subunit embodies the N-glycosidase catalytic activity by removing a specific adenine base from the 28 S rRNA of 60 S ribosomes within infected cells, and hence stop protein synthesis in a targeted cell. The B-subunit binds to the eukaryotic glycolipid receptor globotriaosylceramide (Gb3) or CD77 [3], [4]. There are two major types of Stx designated as Stx1 and Stx2. Stx1 differs at a single amino acid in the A subunit from the Stx of Shigella dysenteriae 1 [5], while Stx2 has approximately 68% and 73% amino acid homology with Stx1 from subunits A and B [6], [31], respectively, and consists of several variants [7]. STEC isolates produce Stx1, Stx2 (or its variants), or both of these toxins. Although the mechanisms of action of the Stxs are thought to be the same, Stx2 is much stronger than Stx1 in mediating HUS [8].

Currently, there is no effective therapy or prophylaxis for HUS other than clinical supportive measures. While certain antibiotics have been shown to increase the risk of HUS development [9], passively administered toxin-specific antibodies have been shown to be highly effective at preventing toxin-mediated diseases. So far, several Stx2-specific monoclonal antibodies have been developed, and many have been shown to neutralize the activity of Stx2 in vitro and/or in vivo [10]–[15], [32]. More recently, a monoclonal antibody (MAb) designated S2C4 has been isolated and shown to be able to neutralize Stx2 and its variant cytotoxicity [34], [35]. It also specifically acts against the A subunit of Stx2 [34], [35]. The availability of Stx2-specific MAb provides an opportunity to administer a safe immunotherapeutic reagent and prevent development of HUS in susceptible individuals.

Understanding how the antibodies recognize their antigens is important for developing antibody therapeutics. Previously, we have developed a new approach for determining the antibody-binding epitope of an antigen [16]. It has been successfully used to identify the important epitopes of the envelop glycoproteins of HIV gp120 to its human neutralizing antibody and to predict the epitopes of ecodomains of glycoproteins of a bunyavirus, “Severe fever with thrombocytopenia syndrome (SFTS) virus”, to its human antibody Mab 4–5 [1]. Briefly, our method involves three steps: Firstly, we identify the locations of chemical functional groups on the key region of the antibody using an exhaustive “multiple copy simultaneous search” (MCSS) approach [17]–[22]. Each of these functional groups corresponds to an individual amino acid type [22]. Secondly, the MCSS clusters of a specific functional group with favorable interaction energies with the protein, also referred to as “minima”, are selected to identify the pattern of functional groups on the surface of the antigen. These functional group patterns are subsequently converted into the amino acid sequence pattern. Thirdly, the antigen protein sequence is sliced into short peptides of seven amino acids, and the set of peptide sequences are scored according to the number of matched amino acids with the sequence pattern identified. The peptides with high score which match the key pattern are considered to be mimotopes. Our method presented here is an extension of our computational combinatorial inhibitor design (CCLD) approach, presented in refs. [19]–[22]. Previously, our CCLD approach has been successfully applied to design peptide inhibitors that could, e.g. block the Ras interacting to its down stream target Raf protein [21], [22]. We developed a novel scheme that allows the application of CCLD to identify several peptide inhibitors that target the protein surface [20]–[22]. Several designed peptides were confirmed by *in vitro* Enzyme-Linked ImmunoSorbant Assay (ELISA), radioassay and Biosensor-based assays [21].

Recently, Smith *et al* studied the recognition regions of Stx2 A subunit to the antibody 11E10 by generating a set of chimeric Stx1/Stx2 molecules and evaluating the capacity of 11E10 to recognize the hybrid toxins using Western Blots and in Vitro cell cytotoxicity assays [23]. Three regions were identified as the epitopes of Stx2 to the antibody; the sequences of these three regions are the most divergent between Stx2 and Stx1 which is why the 11E10 antibody specifically recognizes Stx2 instead of Stx1 [23]. In this work, we will first apply our approach to identify the epitopes of Stx2 subunit A to the 11E10, in comparison with the previous experimental results. Afterwards, we will use it to predict the epitopes of Stx2 subunit A to the newly developed antibody S2C4. The results will be further verified experimentally.

## Methods

### Homology modeling of the antibodies

**Figure 1:**
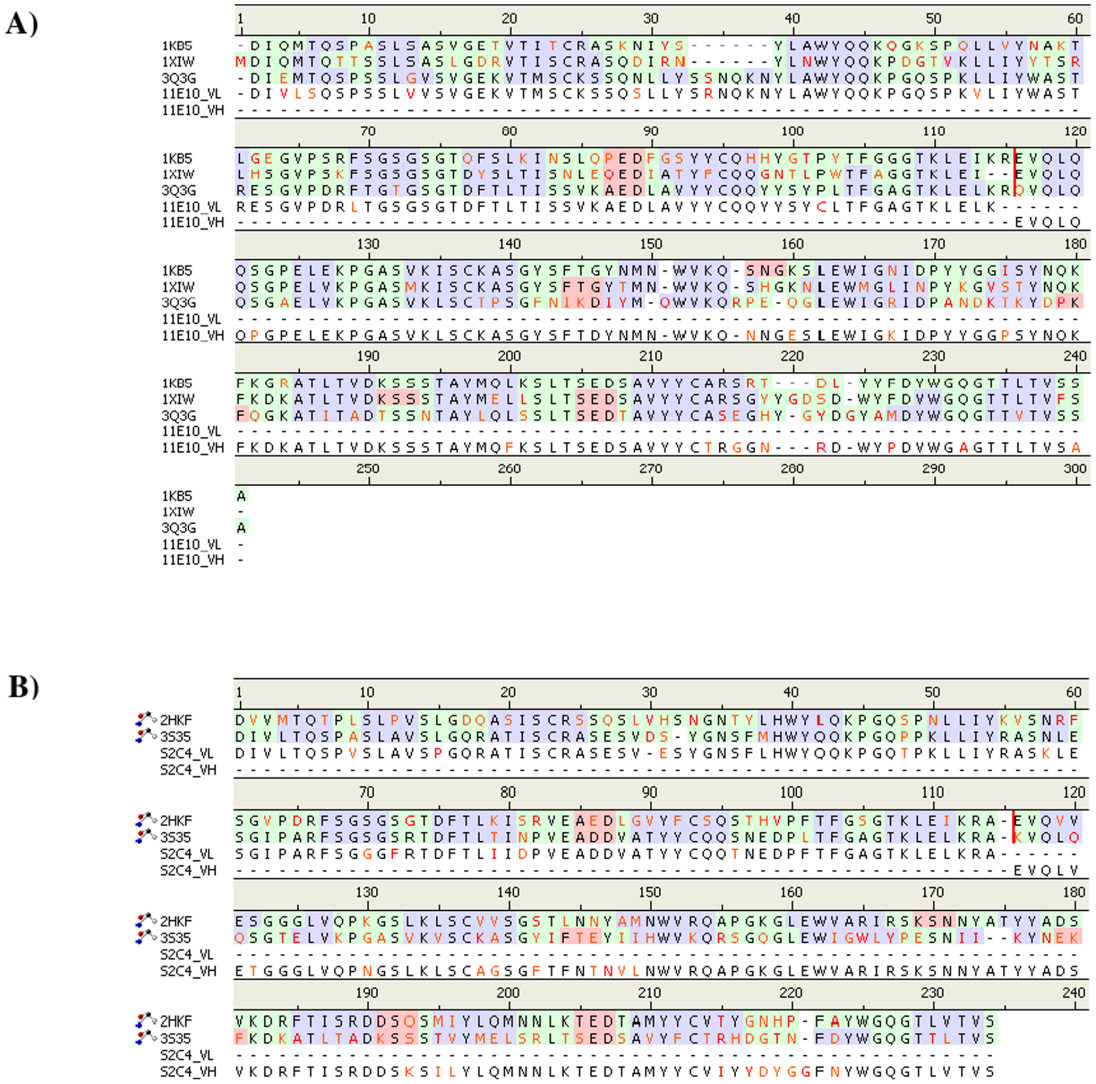
Sequence alignment of the VL and VH domains of the 11E10 and S2C4 antibodies and their templates. A) 11E10 aligned to antibody of the Murine T-cell receptor vairable domain/FAB complex (PDB 1KB5) [24], UCHT1 single-chain antibody fragment (PDB 1XIW) [25] and a Fab fragment of mAb107 complexed to the low- and high-affinity states of CD11bA (PDB 3Q3G) [26]; B) S2C4 aligned to a Murine unglycosylated IgG Fc fragment (PDB 2HKF) [27] and the anti-VEGF receptor antibody IMC-1121B (PDB 3S35) [28].

The sequence of the antibodies 11E10 [33] and S2C4 [34], [35] were used to search for the closest related antibody with known 3D structure using the BLAST (http://blast.ncbi.nlm.nih.gov) database search method focused on sequences of proteins from the protein data bank. Figure 1 shows the sequence alignment of the template antibodies with the VL and VH domains of 11E10 (A) and S2C4 (B). For the 11E10, the templates were found to be PDB entry “1KB5” - Murine T-cell receptor vairable domain/FAB complex [24], entry “1XIW” - UCHT1 single-chain antibody fragment complexed with human CD3-e/d dimer [25] and entry “3Q3G” - Fab fragment of mAb107 complexed to the low- and high-affinity states of CD11bA [26]. The VL and the VH domains of 11E10 show sequence identities of 54% and 84% to 1KB5, 50% and 80% to 1XIW, and 90% and 61% to 3Q3G, respectively. For the S2C4, the best matching antibody sequences found were entry “2HKF” - Murine unglycosylated IgG Fc fragment [27] and entry “3S35” - anti-VEGF receptor antibody IMC-1121B [28]. The amino acid identities of S2C4 are 59% and 81% to 3HKF, 88% and 40% to 3S35 for the VL and VH domains, respectively.

The domains VL and VH of the antibodies 1KB5, 1XIW and 3Q3G PDB structures were used as the templates to create a homology model of the antibody 11E10, and the antibodies 3S35 and 3HKF PDB structures were used to construct the model of the S2C4. The model was created using the Modeller software [29] including explicit optimization of the important loop regions (Complementarity Determining Regions) as implemented in Modeller.

### MCSS of functional groups

The MCSS method has been widely used to determine energetically favorable positions and orientations of functional groups in a target protein [18], [22]. Using the homology model of an antibody, our quCBit software (http://www.computistresearch.com), which implements our MCSS approach, was used to scan the preferred locations of functional chemical groups on the binding surfaces, i.e. the “Complementarity Determining Regions” (CDRs). Eleven functional groups were used, each of which corresponds to the side chains of different amino acids [22], [39]. Table 1 lists the relationship between the functional groups and amino acids. The parameters for both protein and functional groups were taken from the CHARMM22 all-hydrogen atom force field [36].

**Table 1:** The relationship between the functional groups used and amino acids.

	Functional group	Abbreviation	Amino acids
Charged (−)	Acetate ion	ACET	ASP, GLU
Charged (+)	Methylguanidinium	MGUA	ARG
Charged (+)	Methylammonium	MAMM	LYS
Polar	Acetamide	ACEM	ASN,GLN
Polar	Methanol	MEOH	SER,THR
Hydrophobic	Methanethiol	MESH	CYS,MET
Aromatic Polar	Phenol	PHEN	TYR
Aromatic Polar	Indole	INDO	TRP
Aromatic Polar	Imidazole	IMIA	HIS
Aromatic Hydrophobic	Benzene	BENZ	PHE
Hydrophobic	Ibutane	IBUT	VAL, ILE, LEU, ALA

Three hundred replicas of each functional group were randomly distributed inside a sphere with a 12-Å radius around the CDRs of the antibodies. In previous work, we have shown that the details of the CDR loop conformations have insignificant effect on the distribution of MCSS minima and on the sequence pattern derived from the minima [1]. This could be due to the fact that the functional groups are of small size so that the clusters of MCSS minima are insensitive to the conformational change of the CDR loop. Therefore, in this study we use only single conformation of the CDR. The CDRs are defined by (Leu29, Tyr31, Arg33, Trp56, Ser99) of L chain and (Asp31, Asn35, Trp47, Tyr55, Arg57, Gly102) of H chain for the 11E10, and (Val29, Tyr32, Arg54, Glu97) of VL domain and (Thr31, Asn53, Trp47, Asn56, Ala59 and Asp103) of VH domain for the S2C4, respectively. A 500-step multiple copy simultaneous minimisation was performed. During all the MCSS calculations, each replica only interacts with a target protein, and not with the other replicas. The interaction energy, U_MCSS_, was defined as 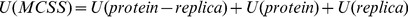
(1)where U(protein-replica) represents non-bonded interactions (i.e. van der Waals and electrostatic interactions) between the target protein and the replica. U(protein_)_ and U(replica) represent the internal energy of the protein and each replica, respectively. In the first protocol where the protein atoms were fixed and each replica treated as a single group, U(protein) and U(replica) were excluded.

The binding energy for a functional group in each minimum obtained from the MCSS calculations was defined as 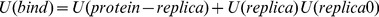
(2)where U(replica0) indicates the internal energy of each replica in vacuum. The nonbonded interaction was truncated at 20 Å. The dielectric constant was set to 10 to mimic solvent screening effects [37].

### Identification of sequence pattern

Interaction energy of −10.00 kcal/mol was used as the threshold for the minima of polar and apolar functional groups, as used in previous work [16]. For the positively charged groups MAMM and MGUA, −10.00 kcal/mol and −15.00 kcal/mol were used for the antibodies 11E10 and S2C4. For the negatively charged group, a threshold of −30.00 kcal/mol was used for the 11E10 and −15.00 kcal/mol for the S2C4, respectively. The larger threshold used for the 11E10 is due to their well-defined electrostatic interactions of the charged residues Arg33 of L chain and Arg102 of H chain of the antibody.

The spatial patterns of the locations of the MCSS minima on the surface of the antibody were converted into a sequence pattern according to the relationship between the functional groups and amino acids as given in Table 1, and this sequence pattern served as the fingerprint to identify the epitopes of antigens.

### Search for epitopes based on the sequence pattern

The sequence pattern obtained using the method described in Section “Identification of sequence pattern” was used to identify the peptides derived from Stx2 subunit A. We divided the whole protein sequence into overlapping peptides of length of seven amino acids as it allows a more efficient scan of the MCSS minima distributions of the average sized binding epitopes. The peptides with a sequence that matched the key pattern derived from MCSS minima of functional groups were considered to be potentially part of the epitope and labeled as the “binders”. To avoid artifacts by starting from a particular residue, the protein was sliced into 7-mer peptide libraries several times, starting from the residue 1 up to 7. This results in seven libraries of 7-mer peptides. Each of the seven libraries was checked for sequence matches with the key pattern. Residues occurring in binder peptides from more than three libraries were considered part of the epitope. Therefore, the epitopes predicted from the seven sets of peptide libraries could vary in their length. The details of the epitope searching were described previously [16].

## Results

### Recognition of Stx2 subunit A to antibody 11E10

**Figure 2:**
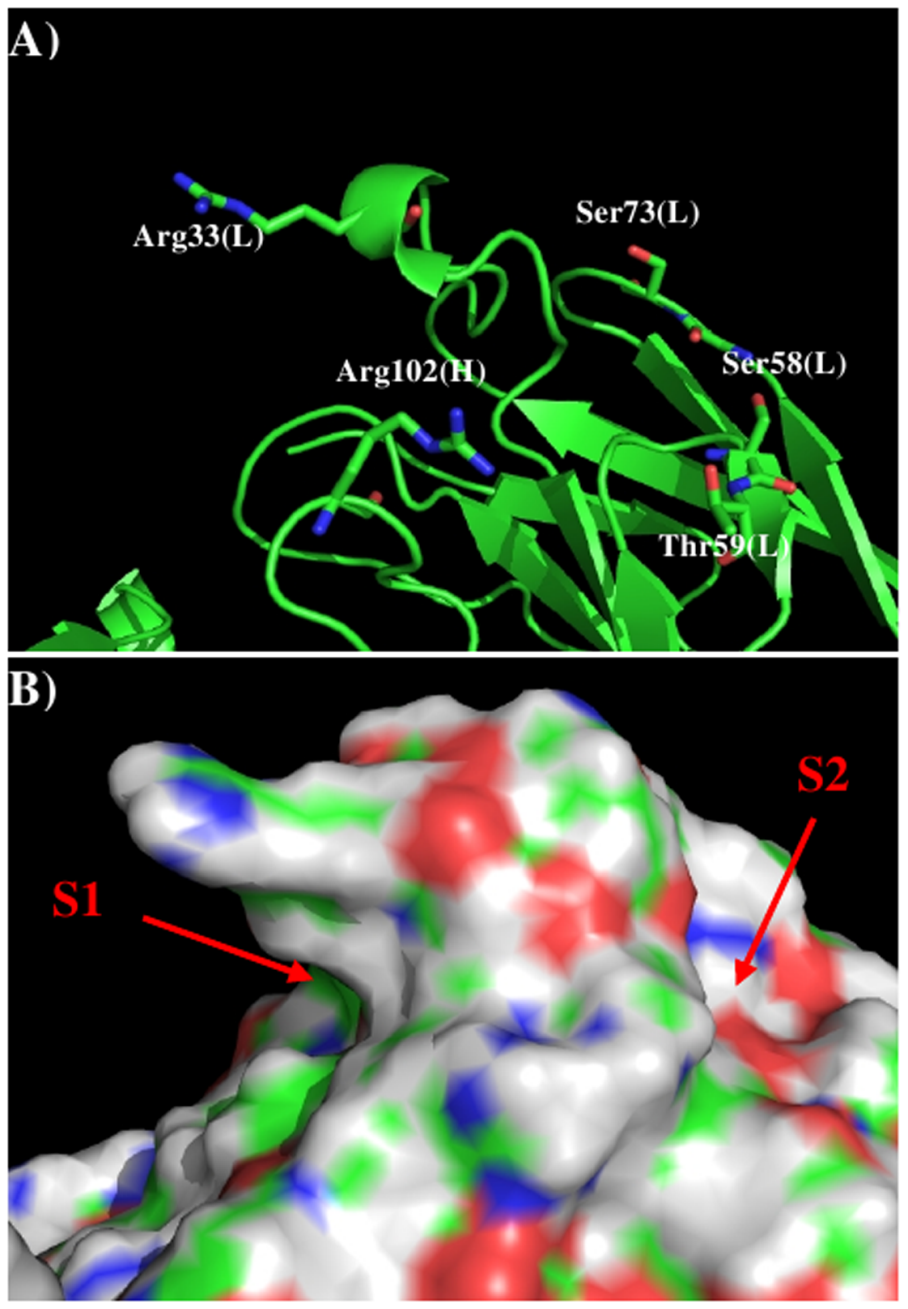
Model structure A) and surface presentation B) of antibody 11E10. The important residues for the antibody interaction are shown in stick form in A). (L) and (H) denote the L and H chain of the antibody respectively. The figure was prepared using PyMOL [38].

Antibody 11E10 has been developed specifically against subunit A of Stx2 and the recognition regions of the Stx2 to the antibody have been investigated experimentally [23]. Firstly we used our approach to identify the epitopes of Stx2 subunit A to 11E10. The antibody structure was built using the X-ray structures with PDB identifiers 1KB5 [24], 1XIW [25] and 3Q3G [26] as templates. Figure 2 shows the model structure and surface of the 11E10 with the important residues highlighted (Figure 2A). Two distinctive regions are identified around the CDR3 loop. Firstly, Arg33 of L chain and Arg102 of H chain form a positively charged surface S1, and secondly, oxygen atoms of Ser58, Thr59 and Ser73 of L chain form a negatively charged surface S2. These two binding surfaces are separated by *ca*. 14.00 Å.

**Figure 3:**
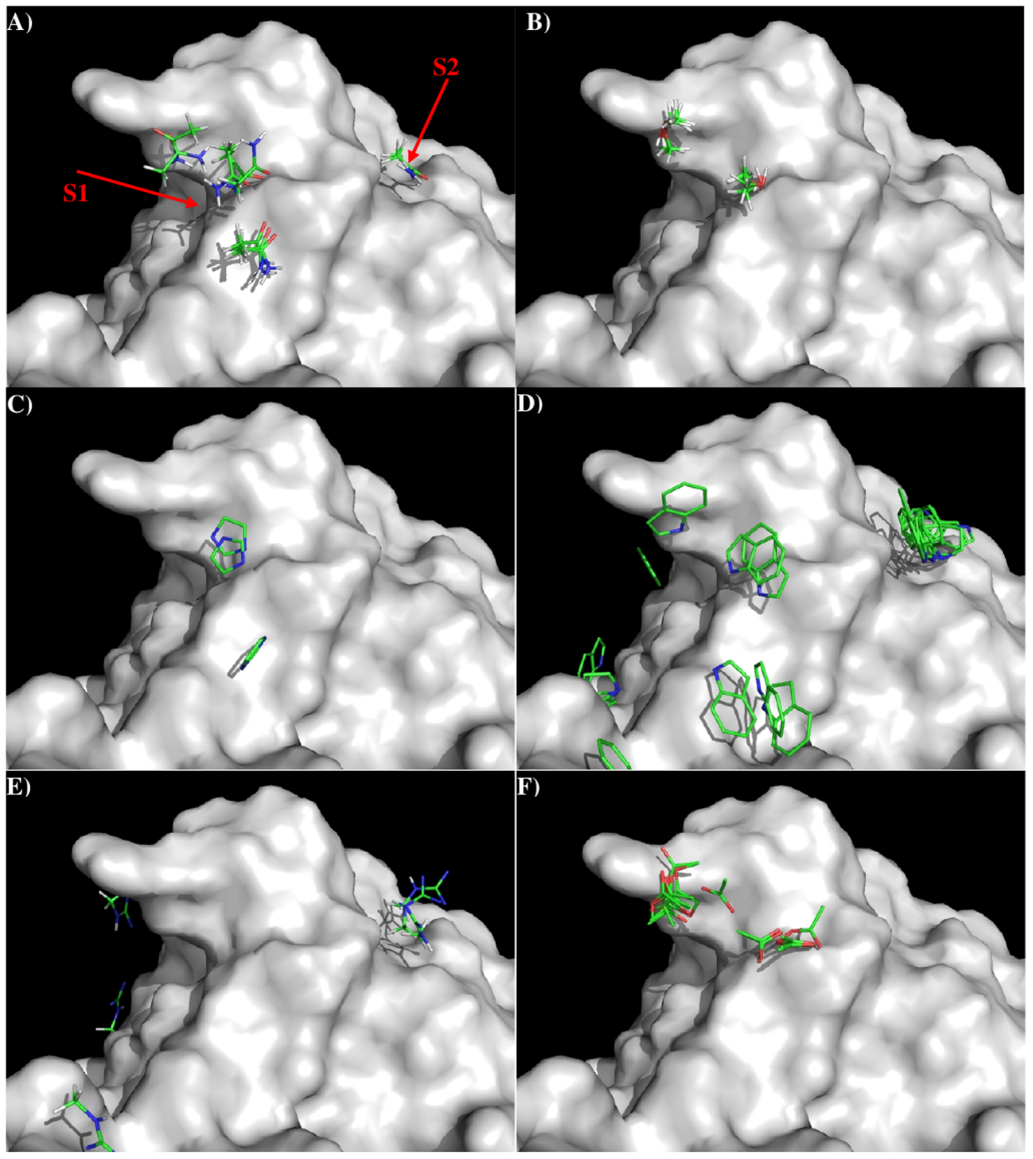
Selected MCSS minima of functional groups on the surface of antibody 11E10. A) ACEM; B) MEOH; C) IMIA; D) INDO; E) MGUA; F) ACET. Figures were prepared using PyMOL[38].

Figure 3 shows the distribution of MCSS minima of functional groups on the surface. Overall, the distributions of the MCSS minima closely correspond to the physical properties. For the apolar groups, MESH and IBUT (small group), BENZ and PHEN (aromatic rings), no minima were found due to the highly charged nature of the surface around the CDR3 loop. In contrast, for the polar groups, ACEM minima were identified with two clusters in S1, forming hydrogen bonds to the charged residues Arg33 of L chain and Arg102 of H chain, respectively. In addition two ACEM minima were also found on S2 with weak interaction energies of −10.00 kcal/mol to residue Thr59 of L chain. The MEOH group showed a strong presence with 42 minima identified in two clusters, interacting with Arg33(L) and Arg102(H), respectively. For the IMIA group, there were three minima interactions with Arg102(H), two of which are hydrogen bonded to Arg102 and the third forms a perpendicular π-π conformation to the side chain of the residue. For the larger INDO group, the majority of minima were located on S2 with the indole-NH atom forming hydrogen bonds to Thr59-OG of the L chain.

For the positively charged groups MAMM, MGUA, and ACET, no MAMM minima were found with interaction energies less than −15.00 kcal/mol. While three MGUA minima were found only on S2 interacting with Thr59(L), eighteen ACET minima were distributed on S1 only and grouped into two clusters interacting with Arg33(L)and Arg102(H), respectively. The best minima were located close to Arg102 with interaction energies of −39.00 kcal/mol.

Using the minima on the two surfaces S1 and S2, we constructed a sequence pattern for the peptides that could potentially bind to the antibody. The maximum distance between the two binding surfaces S1 and S2 is approximately 14.00 Å, corresponding to a separation of three amino acids. While only MGUA minima interact with Thr59 of L chain on the surface S2, the best minima of MEOH, ACEM, IMIA and INDO, as well as ACET interact with both Arg33 of L chain and Arg102 of H chain on S1. Therefore, the key sequence pattern for the binding epitope peptides can be defined as “X—Z”, in which X = R, Z = S,T,Q,N,H or W, and D or E. Note that the sequence is aligned from S2 to S1 as the positively charged minima MGUA is only found on S2 and corresponding to the N-terminal of peptides. Table 2 lists the distribution of key MCSS minima and its derived sequence pattern.

**Table 2:** Distribution of key minima and the derived sequence pattern for the binding epitope peptides to antibody 11E10.

Binding Surface	S2	—14.00 Å—	S1
**MCSS minima Pattern**	MGUA		MEOH
			ACEM
			IMIA
			INDO
			ACET
			
**Sequence Pattern**	R	Gap of 3 amino acid	S/T
			Q/N
			H
			W
			D/E

A sequence pattern of “X—Z” [X = R and Z = (S/T, Q/N, H, W, D/E) ] was obtained.

As the key pattern sequence “X—Z” is derived from the locations of MCSS minima of functional groups corresponding to the side chain of the amino acids, the influence of the spacer region between X and Z on the stability of the peptide structures is expected to be small. To verify this, we constructed the conformations of several peptides of key pattern sequence with randomly selected amino acids for the spacer region. The results showed that the position differences of the key residues X and Z from the different peptides are relatively small with RMSD of less than 0.50 Å.

**Figure 4:**
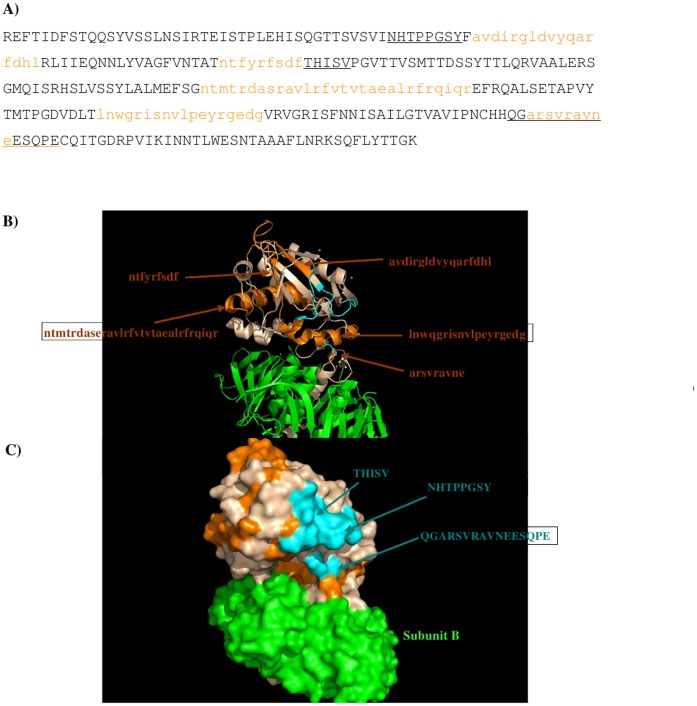
The predicted epitopes of Stx2 to antibody 11E10. A) The predicted epitopes of Stx2 to antibody 11E10 are highlighted in lower case and colored orange in the protein sequence. The recognition regions identified previously (Smith et al 2009) are underlined. B) Backbone presentation of the antigen subunits A and B showing the predicted epitopes in orange and identified regions **A-C** colored in blue. The antibody binds to subunit A only. Subunit B is shown in green. C) Surface presentation of the antigen subunits A and B showing the predicted epitopes in orange and recognition regions **A, B** and **C** colored in blue. Note that the region **C** is only partially shown as the region is missing in the crystal structure of Stx2 (PDB 1R4P) (Fraser et al, 2004). Figures were prepared using PyMOL [38].

The sequence pattern was subsequently used to search for “binders” (see Methods section) from the peptide libraries derived from the sequence of the Stx2 subunit A (given in Figure A in File S1). The seven libraries (see Method section) were searched for peptides matching the calculated sequence pattern of the binding epitope. There are 286 residues in the Stx2 subunit A, which result in 41 peptides of a length of seven amino acids for each set of 7 libraries. Figure B in File S1 shows the identified epitopes from each set of peptide libraries with the epitopes highlighted in orange lower case characters. The five peptides (**1**: residues 51–67, sequence “avdirgldvyqarfdhl”; **2**: residues 87–95, sequence “ntfyrfsdf”; **3**: residues 148–176, sequence “ntmtrdasravlrfvtvtaealrfrqiqr”; **4**: residues 200–217, sequence “lnwgrisnvlpeyrgedg”; **5**: residue 246–254, sequence “arsvravne”) of Stx2 are predicted to bind to the antibody. Figure 4 shows the Stx2 subunit A and subunit B with the epitopes highlighted in lower case and colored in orange (Figure 4A), and their positions in the protein (Figure 4B) and on the protein surface (Figure 4C). The antibody only binds to subunit A. Previously, three regions, i.e. **A** (residues 42–49: NHTPPGSY), **B** (residues 96–101: THISV), and **C** (residue 245–260: QGARSVRAVNESQPE), were identified to be responsible for the specificity of Stx2-11E10 recognition due to the significant sequence divergence at these regions between Stx1 and Stx2 [23]. Figure 4 also shows these regions, as underlined in Figure 4A and colored as cyan in Figures 4B and C. Overall, our calculation reproduced the majority of the region **C** by the binding peptide **5** (“**ARSVRAVNE**” vs “QG**ARSVRAVNE**SQPE”). The binder peptides **1** “AVDIRGLDVYQARFDHL” and **2** “NTFYRFSDF” are adjacent to the regions **A** and **B**, even through peptide **1** is exposed to the solvent on the top of Stx2 subunit A and the majority of peptide **2** is buried inside the protein except the last three amino acids “SDF”. Moreover, peptide **4** is located next to region C and peptide **5**. The recognition surface from regions **A, B** and **C** largely corresponds to the predicted epitope peptides **1** and **5**, and possibly also peptide **4** (Figure 4C). Note that the long peptide **3** forms a helical conformation; it is buried inside the protein and is therefore unable to bind the antibody.

### Prediction of epitopes of Stx2 subunit A to antibody S2C4

**Figure 5:**
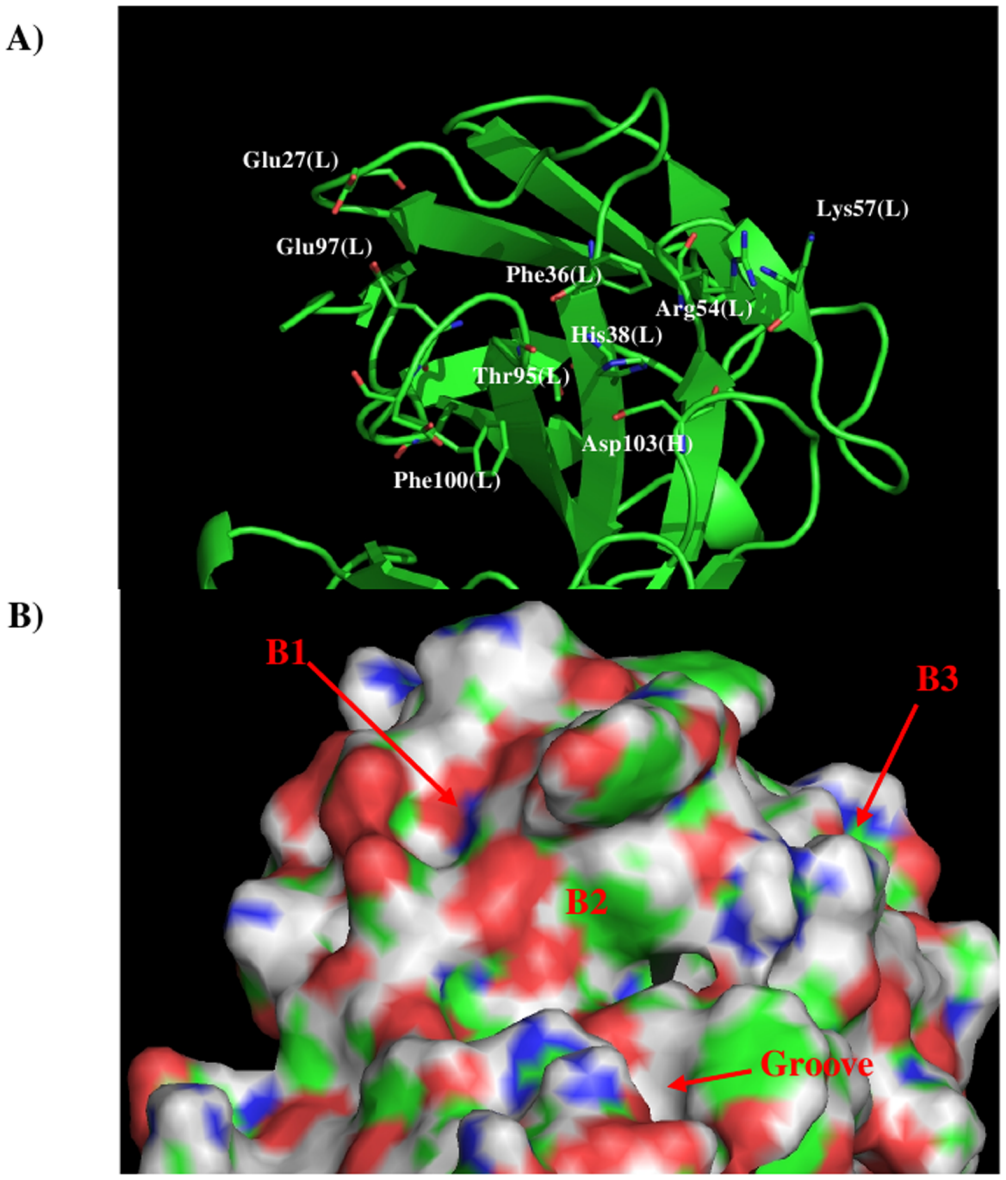
The model structure A) and surface presentation B) of antibody S2C4. The important residues for the antibody interaction are shown in stick form in A). (L) and (H) denote the VL and VH domain of the antibody respectively. The figure was prepared using PyMOL [38].

We created a 3D model of the VL and VH domains of antibody S2C4 using the X-ray structures with PDB identifiers 3HKF [27] and 3S35 [28] as templates. Figure 5 shows the model structure and surface with the important residues highlighted. Compared to the 11E10 antibody, the shape of the surface around the CDR3 loop becomes more defined with a hydrophobic pocket (labeled as B2) formed by residues Phe36, His38, Thr95 of VL domain and Asp103 of VH domain. Two charged binding regions B1 and B3 are formed by residues (Glu27, Glu97) and (Arg54, Lys57) in VL domain, respectively.

**Figure 6:**
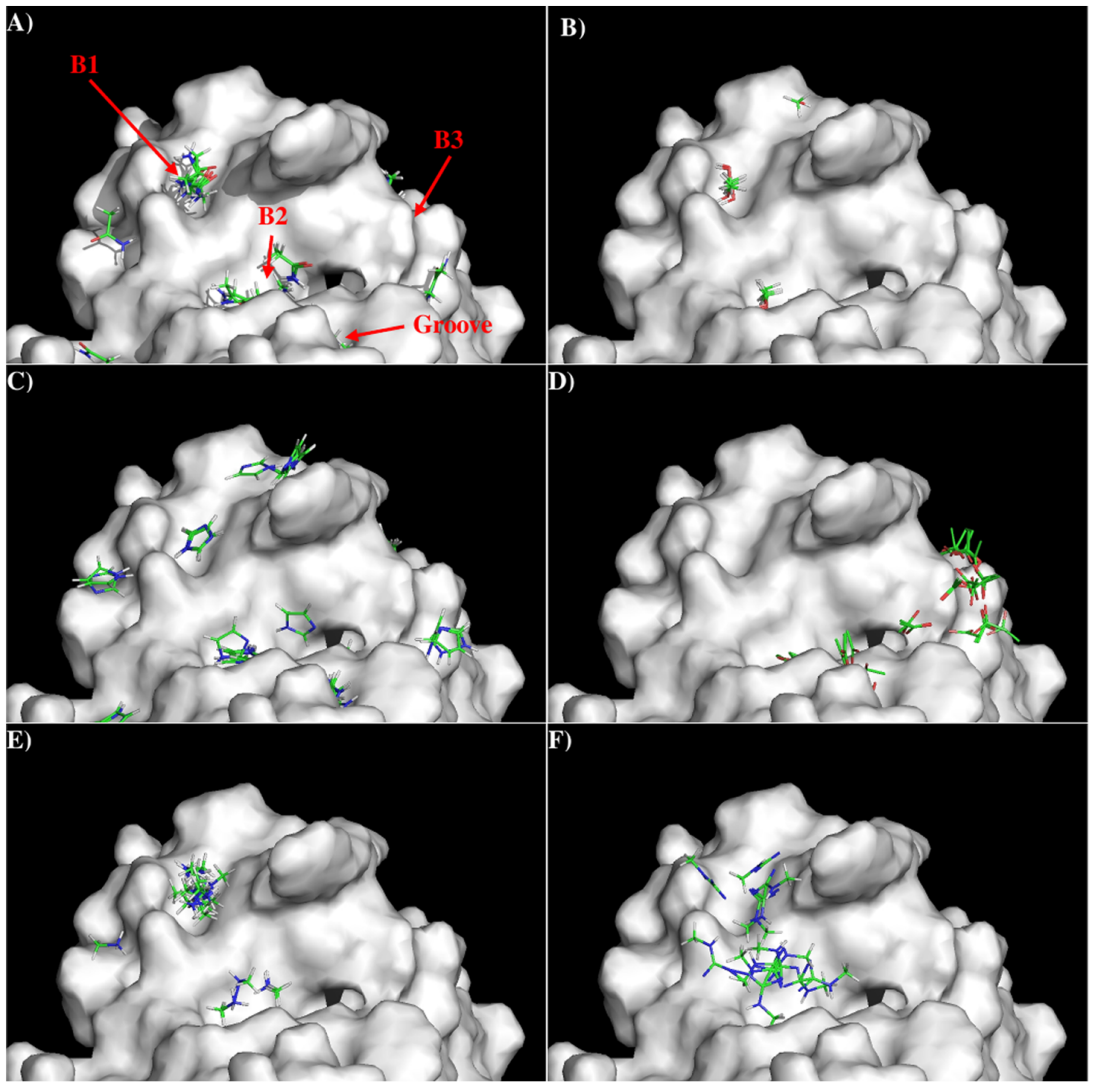
Selected MCSS minima of functional groups on the surface of antibody S2C4 against subunit A of Stx2. A) ACEM; B) MEOH; C) IMIA; D) ACET; E) MAMM; F) MGUA. Figures were prepared using PyMOL [38].

Figure 6 shows the locations of the minima of functional groups on the surface of S2C4. Overall, the minima are clustered around the three binding sites B1, B2 and B3 as described above, except that some minima are located inside a narrow groove formed between side chains of peptides Arg50-Asp56 and Asp103-Tyr104 of VH domain. Due to the flexibility of the side chains (especially the turn at residues 103–104 of the VH domain), this groove is unstable. Therefore, we disregarded the minima located inside the groove and focused on those at the binding sites B1, B2 and B3. Overall, no minima of apolar groups such as MESH, IBUT, BENZ and PHEN were found on the surfaces around the CDR3 loop of the S2C4. For the polar groups, ACEM minima were found at the three binding sites – clusters of 22, 20 and 7 with favorable interaction energy values of −11.10 kcal/mol (B1), −13.90 kcal/mol (B2) and −12.20 kcal/mol (B3), respectively. Twenty five MEOH minima were located at B1 and B2 with the best minima at B2. In the aromatic group, IMIA minima were distributed over the surface with 36, 12 and 7 minima located at the three binding sites. The best minima interactions involved Glu27 and Glu97 at B1, Asp98 and Phe100 at B2, and Arg54 at B3, with the binding energies of −11.80 kcal/mol, −12.00 kcal/mol and −12.40 kcal/mol, respectively. For the positively charged groups MAMM and MGUA, 27 MAMM minima were concentrated at B1 through electrostatic interactions to Glu27 and Glu97, and 4 minima were inside the pocket B2 held in place by hydrogen bonding to the carbonyl oxygen of Thr95 and Asn96. However, the 13 MGUA minima identified were spread over B1 and B2 with the best minima at B1 having an interaction energy of −18.20 kcal/mol. For the negatively charged group ACET, 25 minima were distributed at the B3 site by forming salt bridges to Arg54 or Lys57.

Based on the distribution of the important minima as shown in Figure 6, a sequence pattern for peptides that bind to S2C4 was derived. The MCSS minima at the binding sites B2 and B3 are separated by ca 10.50 Å, a distance that could accommodate two amino acids. While only ACET minima were obtained at B3 exhibiting interactions with Arg54 and Lys57 of VL domain, most of the functional groups are located in the sites B1 and B2. At B1, the positively charged groups MGUA and MAMM showed the best minima with strong interaction energies, due to the strong electrostatic interactions to the negatively charged residues Glu27 and Glu97 of VL domain. Polar groups are mainly located inside the binding pocket at B2, however, because B1 and B2 are ca. 5.00 Å apart there is still a significant preference for MGUA and MAMM at B1. Therefore, the key sequence pattern for the binders was defined as “XZ—J”, in which X = R or K, Z = R, Q or N, H, S or T, and J = D or E. Table 3 lists the distribution of key MCSS minima and the derived sequence pattern. Due to the short length of peptides (7mer), we loosened the search criteria to “XZ” only. A similar approximation has been applied previously [16].

**Table 3:** Distribution of key minima and the derived sequence pattern for the binding epitope peptides to the antibody S2C4.

Binding Site	B1	B2	—10.50 Å—	B3
**MCSS minima Pattern**	MGUA	MEOH		ACET
	MAMM	ACEM		
		IMIA		
		MGUA		
				
**Sequence Pattern**	R	S/T	Gap of 2 amino acid	D/E
	K	Q/N		
		H		
		R		
		S/T		
				

A sequence pattern of “XZ—J”(X = R or K, and Z = R, Q or N, H, S or T, and J = D or E) was obtained.

**Figure 7:**
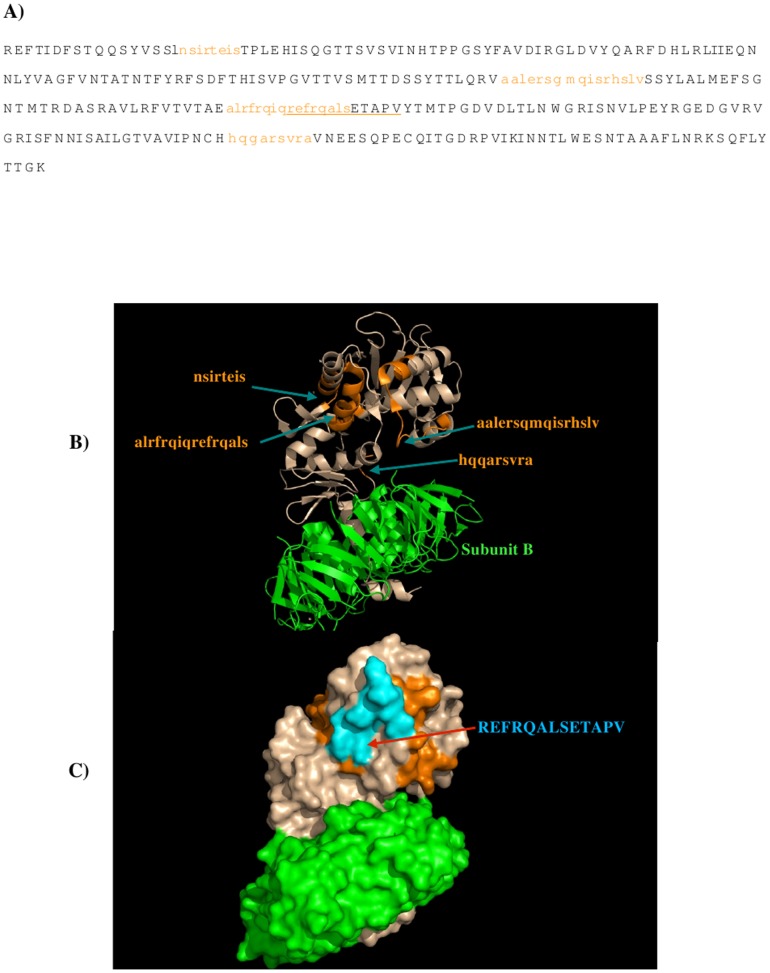
The predicted epitopes of Stx2 to antibody S2C4. A) The predicted epitopes of Stx2 to antibody S2C4 are highlighted in lower case and colored orange in the protein. The recognition regions identified previously (Fraser et al, 2004) are underlined. B) Backbone presentation of the antigen subunits A and B showing the predicted epitopes on subunit A in orange. The antibody is predicted to bind only to subunit A. C) Surface presentation of the antigen subunits A and B showing the predicted epitopes on subunit Ain orange and a novel recognition region **D** colored in cyan. Note that the region **C** is only shown partially as the region is missed in the crystal structure of Stx2 (PDB 1R4P) (Fraser et al, 2004). Figures were prepared using PyMOL [38].

**Figure 8:**
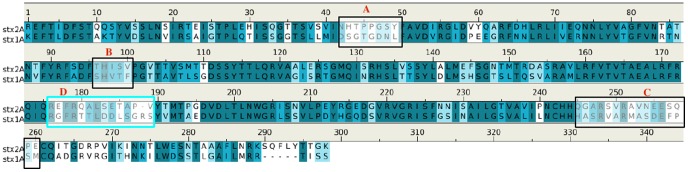
Sequence alignment of subunit A of Stx2 and the subunit A of Stx1. The boxes highlight the recognition regions responsible for the selectivity of antibodies 11E10 and S2C4 for the subunit A of Stx2 only. Regions **A, B** and **C** are indicated by a black box, and the novel recognition region **D** identified here to be responsible for the selectivity of the S2C4 is highlighted by a cyan box.

Seven sets of peptide libraries were generated using the protocol as described in Method section “Search for epitopes based on the sequence pattern”. Figure C in File S1 show the identified binders of each set of peptide libraries with the binder residues highlighted in lower case and colored orange. The final predicted epitopes of the Stx2 subunit A using the protocol described in section “Search for epitopes based on the sequence pattern” are shown in Figure 7A) with the epitope shown in orange lower case characters. We number the epitopes as **I-IV** so as to distinguish them from the epitopes (**1–5**) predicted for antibody 11E10Overall, four binding peptides (**I**: residue 18–25, sequence “NSIRTEIS”; **II**: residues 121–136, sequence “AALERSGMQISRHSLV”; **III**: residues 168–183, sequence “ALRFRQIQREFRQALS”; **IV**: residues 243–251, sequence “HQGARSVRA”) are predicted and these epitopes are located on the surface of subunit A of Stx2. If we compare these Stx2 epitopes to the experimentally derived recognition regions and the predicted epitopes of the 11E10 (Figure 4), significant overlap can be observed in the two antibodies. Stx2 shares the recognition region **C** - overlap occurs in both the predicted epitope of peptide **5** to the 11E10 and peptide **IV** to the S2C4. However, the binding peptides **I-III** are located on the opposite side of the recognition regions (epitopes) to the 11E10 (Figure 7B), due to the different sequence pattern derived from MCSS minima. Interestingly, the peptides **II** and **III** are located closely enough to form a recognition surface which on examination contains the sequence that is least conserved between Stx1 and Stx2. This region is located between binding peptides **II** and **III**, and labeled as **D** (residues 176–188, sequence “REFRQALSETAPV”, as colored in cyan in Figure 7C). The significant sequence differences of region **D** between Stx1 and Stx2 (Figure 8), give a strong indication that this is a novel recognition region, resulting in the selective binding of the antibody S2C4 to the Stx2.

## Discussion

Previously, we have developed a new method to predict the binding epitopes of an antigen to the antibody [16]. It involves three steps: 1) mapping of functional groups onto the surface of the antibody, 2) deriving a sequence pattern for potential binding peptides based on the distribution of significant minima of functional groups, and 3) searching the binding peptides from the sequence of the antigen. Here, we have applied the new method to identify binding epitopes of Shiga Toxin to two independent antibodies 11E10 [23], [30] and S2C4 [34], [35] that have been developed specifically against the subunit A of Stx2. Our results provide insights into the recognition mechanisms of Stx2 to the antibodies. Our method is able to predict and identify a set of peptides that potentially form part of the epitope, thus significantly reducing the amount of experimental work needed to find an antibody binding epitope.

For the epitopes of Stx2 binding to its specific antibody 11E10, experimental work has identified three recognition regions **A, B** and **C** in the subunit A of Stx2 [23] (as shown in Figure 4). Based on the model structure of the 11E10, the MCSS calculations predicted five peptides as potential candidates for the binding epitopes (Figure 4). One of the peptides “ARSVRAVNE” is in fact consistent to the region **C** identified experimentally [23]. While the epitope peptides “AVDIRGLDVYQARFDHL” and “NTFYRFSDF” are located next to the regions **A** and **B**, respectively, they are either exposed away from the recognition surface or buried inside the protein (Figure 4). Nevertheless, the predicted epitopes “NTMRDASRAVLRFVTVTAEALRFRQIQR”, “LNWGRISNVLPEYRGEDG” and “ARSVRAVNE” are close enough to resemble the recognition surface constructed by regions **A, B** and **C** (Figure 4). Our calculations thus qualitatively reproduced the experimental recognition regions, and demonstrate a significant simplification of the experimental search for the binding epitope. In addition, the MCSS minima are grouped around the charged residues Arg33 of L chain and Arg102 of H chain of the antibody due to a flat surface around the CDR3 loop of 11E10 (Figure 3). This indicates that the antibody-antigen recognition of Stx2 and 11E10 is determined by these charged residues.

For the antibody S2C4, the surface of antibody around the CDR3 loop becomes more defined with a hydrophobic pocket formed by residues Phe36, His38, Thr95 of VL domain and Asp103 of VH domain (Figure 5). The distribution of MCSS minima are thus different from those found in 11E10 (Figure 6 vs Figure 3). Accordingly, the sequence pattern identified from the MCSS minima is significantly different (Table 2 vs Table 3). Four peptides are predicted as the binding epitopes of the S2C4. While the peptide “HQGARSVRA” overlays with the recognition region **C** of Stx2 subunit A that binds the antibody 11E10, the other three epitopes are located on the region opposite to the epitopes of 11E10, indicating different recognition regions for the two antibodies (Figure 7 vs Figure 4).

Both antibodies selectively bind to Stx2. For the 11E10, experimental work showed that the selectivity is due to its binding to the three epitope regions **A**, **B** and **C** in which the sequence of Stx2 is divergent from Stx1 as shown in Figure 8 [23], and our calculations are consistent with the experimental observations. For the antibody S2C4, our results suggest that selectivity is achieved by binding to the epitope “HQGARSVRA” which is similar to the region **C.** However, our results also suggest the possibility of an alternative site, that is, to the epitopes “AALERSGMQISRHSLV” and “ALRFRQIQREFRQALS” which form a separate recognition surface (Figure 7). This is of particular interest because the predicted epitope “ALRFRQIQ**REFRQALS**” contains a region (residues 176–188, sequence “**REFRQALS**ETAPV”) where the sequence is significantly different between Stx1 and Stx2, presenting a new epitope region (region **D**) for selective antibody recognition (Figure 8).

## Conclusions

Previously we have developed a simple qualitative method to search the epitopes of the antigen that bind to an antibody [16]. In this work, we have applied this method to identify the antibody recognition regions of Stx2 subunit A to the antibodies 11E10 and S2C4. Both antibodies bind selectively to the Stx2 of the Shiga Toxin family, as illustrated by the predicted epitopes from our calculations. For the 11E10, the possible epitopes are “AVDIRGLDVYQARFDHL”, “NTFYRFSDF”, “LNWGRISNVLPEYRGEDG” and “ARSVRAVNE” which form the recognition surfaces incorporated in the regions of NHTPPGSY (**A**), THISV (**B**), QGARSVRAVNEESQPE (**C**) identified experimentally [23]. The recognition regions NHTPPGSY and QGARSVRAVNEESQPE are the least conserved regions of Stx2 and other Stxs, and are responsible for the observed selectivities of the 11E10 [23]. For the S2C4, the best epitopes are predicted to be residues 121–136 (sequence “AALERSGMQISRHSLV”), 168–183 (seqeuence “ALRFRQIQREFRQALS”, and 243–251 (sequenece “HQGARSVRA”). While the third epitope overlays with the observed recognition region **C**, the first two epitopes indicate a novel recognition region **D** (residues 176–188, sequence “REFRQALSETAPV”) with significant sequence difference between Stx2 and other Shiga toxins. Therefore, there are strong indications that S2C4 specifically binds to the Stx2 subunit A at either the region **C** and/or the region **D**. The shared recognition region **C** between two antibodies and the novel region **D** of antibody S2C4 to the Shiga Toxin 2 will be tested using an in vitro binding assay to verify our prediction.

## Supporting Information

File S1 **Figure A-C.** Figure A: Sequence of Shiga toxin 2 (Stx2) Subunit A. Figure B: Sequence alignment of Stx2 Subunit A with 7 sets of binder to antibody 11E10 highlighted in orange lower case characters. Figure C: Sequence alignment of Stx2 Subunit A with 7 sets of binder to antibody S2C4 highlighted in orange lower case characters.
(DOC)
