# Supplementary material for: Antibody Recognition of Shiga Toxins (Stxs): Computational Identification of the Epitopes of Stx2 Subunit A to the Antibodies 11E10 and S2C4
Source: PLoS One. 2014 Feb 5;9(2):e88191. doi: 10.1371/journal.pone.0088191 (PMC3917601; doi:10.1371/journal.pone.0088191)
Supplement: File S1 — Figure A-C. Figure A: Sequence of Shiga toxin 2 (Stx2) Subunit A. Figure B: Sequence alignment of Stx2 Subunit A with 7 sets of binder to antibody 11E10 highlighted in orange lower case characters. Figure C: Sequence alignment of Stx2 Subunit A with 7 sets of binder to antibody S2C4 highlighted in orange lower case characters. (DOC) [file pone.0088191.s001.doc]

**Antibody Recognition Regions of Shiga Toxins (Stxs):**

**Computational Identification of the Epitopes of Stx2 Subunit A to the Antibodies 11E10 and S2C2.**

Yongjun Jiao^1^, Sue F. Legge^3^, Xiaoyan Zeng^1^, Herbert R. Treutlein^2,3*^ and Jun Zeng^2,3^*

1. Institute of Pathogenic Microbiology, Jiangsu Provincial Center for Disease Prevention and Control, Key Laboratory of Enteric Pathogenic Microbiology, Ministry Health, Nanjing 210009, China
2. Monash Institute of Pharmaceutical Sciences (MIPS), Monash University, Parkville VIC 3050, Australia
3. Computist Bio-Nanotech, Small Technology Cluster, Scoresby, VIC 3179, Australia

*Corresponding authors.

Jun Zeng Tel: +61 4 13323321, Email: [Jun.Zeng@computistresearch.com](mailto:Jun.Zeng@computistresearch.com), Herbert Treutlein: +61 412 367 935, email: [Herbert.Treutlein@computistresearch.com](mailto:Herbert.Treutlein@computistresearch.com).

**Supplementary Materials**

REFTIDFSTQQSYVSSLNSIRTEISTPLEHISQGTTSVSVINHTPPGSYFAVDIRGLDVYQARFDHLRLIIEQNNLYVAGFVNTATNTFYRFSDFTHISVPGVTTVSMTTDSSYTTLQRVAALERSGMQISRHSLVSSYLALMEFSGNTMTRDASRAVLRFVTVTAEALRFRQIQREFRQALSETAPVYTMTPGDVDLTLNWGRISNVLPEYRGEDGVRVGRISFNNISAILGTVAVIPNCHHQGARSVRAVNEESQPECQITGDRPVIKINNTLWESNTAAAFLNRKSQFLYTTGK

**Figure A: Sequence of Shiga toxin 2 (Stx2) Subunit A**

1.reftidfSTQQSYVSSLNSIRTEISTPLEHISQGTTSVSVINHTPPGSYfavdirgldvyqarFDHLRLIIEQNNLYVAGFVNTatntfyrFSDFTHIS

2.REFTIDFSTQQSYVSSLNSIRTEISTPLEHISQGTTSVSVINHTPPGSYFavdirgldvyqarfDHLRLIIEQNNLYVAGFVNTAtntfyrfSDFTHIS

3.REFTIDFSTQQSYVSSLNSIRTEISTPLEHISQGTTSVSVINHTPPGSYFAvdirgldvyqarfdHLRLIIEQNNLYVAGFVNTATntfyrfsDFTHIS

4.REFTIDFSTQQSYVSSLNSIRTEISTPLEHISQGTTSVSVINHTPPGSYFAVdirgldvyqarfdhLRLIIEQNNLYVAGFVNTATNtfyrfsdFTHIS

5.REFTIDFSTQQSYVSSLNSIRTEISTPLEHISQGTTSVSVINHTPPGSYFAVDirgldvyqarfdhlRLIIEQNNLYVAGFVNTATNTfyrfsdfTHIS

6.REFTIDFSTQQSYVSSLNSIRTEISTPLEHISQGTTSVSVINHTPPGSYFAVDIrgldvyqarfdhlrLIIEQNNLYVAGFVNTATNTFyrfsdftHIS

7.REFTIDFSTQQSYVSSLNSIRTEISTPLEHISQGTTSVSVINHTPPGSyfavdirGLDVYQArfdhlrlIIEQNNLYVAGFVNTATNTFYrfsdfthIS

1.VPGVTTVSMTTDSSYTTLQRVAALERSGMQISRHSLVSSYLALMEFSGntmtrdasravlrfVTVTAEAlrfrqiqREFRQALSETAPVYTMTPGDVDL

2.VPGVTTVSMTTDSSYTTLQRVAALERSGMQISRHSLVSSYLALMEFSGNtmtrdasravlrfvTVTAEALrfrqiqrEFRQALSETAPVYTMTPGDVDl

3.VPGVTTVSMTTDSSYTTLQRVAALERSGMQISRHSLVSSYLALMEFSGNTmtrdasravlrfvtvtaealrfrqiqreFRQALSETAPVYTMTPGDVDL

4.VPGVTTVSMTTDSSYTTLQRVAALERSGMQISRHSLVSSYLALMEFSGNTMtrdasravlrfvtvtaealrfrqiqrefRQALSETAPVYTMTPGDVDL

5.VPGVTTVSMTTDSSYTTLQRVAALERSGMQISRHSLVSSYLALMEFSGNTMTrdasravlrfvtvtaealrfrQIQREFRQALSETAPVYTMTPGDVDL

6.VPGVTTVSMTTDSSYTTLQRVAALERSGMQISRHSLVSSYLALMEFsgntmtrDASRAVLrfvtvtaealrfrqIQREFRQALSETAPVYTMTPGDVDL

7.VPGVTTVSMTTDSSYTTLQRVAALERSGMQISRHSLVSSYLALMEFSgntmtrdasravlrFVTVTAEalrfrqiQREFRQALSETAPVYTMTPGDVDL

1.TLNWGrisnvlpeyrgedgVRVGRISFNNISAILGTVAVIPNCHHQGarsvravNEESQPECQITGDRPVIKINNTLWESNTAAAFLNRKSQFLYTTGK

2.tlnwgrISNVLPEyrgedgvRVGRISFNNISAILGTVAVIPNCHHQGArsvravnEESQPECQITGDRPVIKINNTLWESNTAAAFLNRKSQFLYTTGK

3.tlnwgriSNVLPEYrgedgvrVGRISFNNISAILGTVAVIPNCHHQGARsvravneESQPECQITGDRPVIKINNTLWESNTAAAFLNRKSQFLYTTGK

4.TlnwgrisnvlpeyrGEDGVRVGRISFNNISAILGTVAVIPNCHHQGARSvravneeSQPECQITGDRPVIKINNTLWESNTAAAFLNRKSQFLYTTGK

5.TLnwgrisnvlpeyrgEDGVRVGRISFNNISAILGTVAVIPNCHHQGARSVravneesQPECQITGDRPVIKINNTLWESNTAAAFLNRKSQFLYTTGK

6.TLNwgrisnvlpeyrgeDGVRVGRISFNNISAILGTVAVIPNCHHqgarsvrAVNEESQPECQITGDRPVIKINNTLWESNTAAAFLNRKSQFLYTTGK

7.TLNWgrisnvlpeyrgedGVRVGRISFNNISAILGTVAVIPNCHHQgarsvraVNEESQPECQITGDRPVIKINNTLWESNTAAAFLNRKSQFLYTTGK

**Figure B: Sequence alignment of Stx2 Subunit A with 7 sets of binder to antibody 11E10 highlighted in orange lower case characters.**

1.REFTIDFSTQQSYVsslnsirTEISTPLEHISQGTTSVSVINHTPPGSYFAVDIRGLDVYQARFDHLRLIIEQNNLYVAGFVNTATNTFYRFSDFTHIS

2.REFTIDFSTQQSYVSslnsirtEISTPLEHISQGTTSVSVINHTPPGSYFAVDIRGLDVYQARFDHLRLIIEQNNLYVAGFVNTATNTFYRFSDFTHIS

3.REFTIDFSTQQSYVSSlnsirteISTPLEHISQGTTSVSVINHTPPGSYFAVDIRGLDVYQARFDHLRLIIEQNNLYVAGFVNTATNTFYRFSDFTHIS

4.REFTIDFSTQQSYVSSLnsirteiSTPLEHISQGTTSVSVINHTPPGSYFAVDIRGLDVYQARFDHLRLIIEQNNLYVAGFVNTATNTFYRFSDFTHIS

5.REFTIDFSTQQSYVSSLNsirteisTPLEHISQGTTSVSVINHTPPGSYFAVDIRGLDVYQARFDHLRLIIEQNNLYVAGFVNTATNTFYRFSDFTHIS

6.REFTIDFSTQQSYVSSLNSirteistPLEHISQGTTSVSVINHTPPGSYFAVDIRGLDVYQARFDHLRLIIEQNNLYVAGFVNTATNTFYRFSDFTHIS

7.REFTIDFSTQQSYVSSLNSIrteistpLEHISQGTTSVSVINHTPPGSYFAVDIRGLDVYQARFDHLRLIIEQNNLYVAGFVNTATNTFYRFSDFTHIS

1.VPGVTTVSMTTDSSYTTLQRvaalersgmqisrhSLVSSYLALMEFSGNTMTRDASRAVLRFVTVTAEAlrfrqiqrefrqalSETAPVYTMTPGDVDL

2.VPGVTTVSMTTDSSYTTLQRVaalersgmqisrhsLVSSYLALMEFSGNTMTRDASRAVLRFVTVTAEALrfrqiqrefrqalsETAPVYTMTPGDVDL 3.VPGVTTVSMTTDSSYTTLQRVAalersgmqisrhslVSSYLALMEFSGNTMTRDASRAVLRFVTVTAEALRfrqiqrefrqalseTAPVYTMTPGDVDL 4.VPGVTTVSMTTDSSYTTLQRVAAlersgmqisrhslvSSYLALMEFSGNTMTRDASRAVLRFVTVTAEALRFrqiqrefrqalsetAPVYTMTPGDVDL 5.VPGVTTVSMTTDSSYTTLQRVAALersgmqisrhslvsSYLALMEFSGNTMTRDASRAVLRFVTVTaealrfrqiqrefrQALSETAPVYTMTPGDVDL 6.VPGVTTVSMTTDSSYTTLQRVAALErsgmqisrhslvssYLALMEFSGNTMTRDASRAVLRFVTVTAealrfrqiqrefrqALSETAPVYTMTPGDVDL 7.VPGVTTVSMTTDSSYTTLQrvaalersgmqisrHSLVSSYLALMEFSGNTMTRDASRAVLRFVTVTAEalrfrqiqrefrqaLSETAPVYTMTPGDVDL

1.TLNWGRISNVLPEYRGEDGVRVGRISFNNISAILGTVAVIPNCHHQGarsvravNEESQPECQITGDRPVIKINNTLWESNTAAAFLNRKSQFLYTTGK

2.TLNWGRISNVLPEYRGEDGVRVGRISFNNISAILGTVAVIPNCHHQGArsvravnEESQPECQITGDRPVIKINNTLWESNTAAAFLNRKSQFLYTTGK 3.TLNWGRISNVLPEYRGEDGVRVGRISFNNISAILGTVAVIPNchhqgarSVRAVNEESQPECQITGDRPVIKINNTLWESNTAAAFLNRKSQFLYTTGK 4.TLNWGRISNVLPEYRGEDGVRVGRISFNNISAILGTVAVIPNChhqgarsVRAVNEESQPECQITGDRPVIKINNTLWESNTAAAFLNRKSQFLYTTGK 5.TLNWGRISNVLPEYRGEDGVRVGRISFNNISAILGTVAVIPNCHhqgarsvRAVNEESQPECQITGDRPVIKINNTLWESNTAAAFLNRKSQFLYTTGK 6.TLNWGRISNVLPEYRGEDGVRVGRISFNNISAILGTVAVIPNCHHqgarsvrAVNEESQPECQITGDRPVIKINNTLWESNTAAAFLNRKSQFLYTTGK 7.TLNWGRISNVLPEYRGEDGVRVGRISFNNISAILGTVAVIPNCHHQgarsvraVNEESQPECQITGDRPVIKINNTLWESNTAAAFLNRKSQFLYTTGK

**Figure C: Sequence alignment of Stx2 Subunit A with 7 sets of binder to antibody S2C2 highlighted in orange lower case characters.**
